# Triple A Multisystem Disorder: Allgrove Syndrome

**DOI:** 10.7759/cureus.17476

**Published:** 2021-08-26

**Authors:** Mohamad Alhassoun, Abdul Hakim Almakadma, Sami Almustanyir, Abed AlLehibi, Nawaf Alotaibi

**Affiliations:** 1 College of Medicine, Alfaisal University, Riyadh, SAU; 2 Internal Medicine, Ministry of Health, Riyadh, SAU; 3 Gastroenterology, King Fahad Medical City, Riyadh, SAU

**Keywords:** allgrove syndrome, alacrimia, achalasia, adrenocorticotrophic resistant adrenal insufficiency, aaas gene

## Abstract

Allgrove syndrome (AS), also known as triple A syndrome, is an autosomal recessive inherited disorder characterized by a triad of alacrimia, achalasia, and adrenocorticotrophic hormone (ACTH) resistant adrenal insufficiency. We present a case of a 16-year-old male presenting with the classic triad and a homozygous mutation in the Aladin WD Repeat Nucleoporin (AAAS gene). Genetic analysis and detection of AAAS gene mutation is the cornerstone of diagnosis. Delayed detection results in multiple hospital admissions and life-threatening complications. More data and research are needed to aid in expanding the knowledge about Allgrove syndrome in the literature.

## Introduction

Allgrove syndrome (AS), also known as triple A syndrome, is an autosomal recessive inherited disorder first reported in 1978 by Allgrove et al. [1]. AS is characterized by a triad of alacrimia, achalasia, and adrenocorticotrophic hormone (ACTH) resistant adrenal insufficiency. However, other symptoms such as neurological deficits and mild mental retardation are also associated with the disorder [2]. Onset of symptoms is usually seen before the age of 10; however, adulthood cases have been reported [3].

Diagnosis of AS is often most reliably confirmed by genetic sequencing in suspected individuals. However, diagnosis is often challenging without the classic clinical presentation [4]. Interventions in AS are usually dependent on the case’s severity and include glucocorticoids, artificial tears eyedrops, and esophageal procedures such as balloon pneumatic dilatation or peroral endoscopic myotomy (POEM) [5,6]. 

## Case presentation

We present a case of a 16-year-old male with a known case of AS, diagnosed a year ago. The diagnosis was confirmed by a positive gene study of homozygous mutation is AAAS gene as well as clinical manifestations of achalasia, alacrimia, and partial adrenal insufficiency. Our patient had an extensive history of multiple hospital visits with vague symptoms resulting in delayed diagnosis. The symptoms included difficulty swallowing, chronic diarrhea, abdominal pain, and vomiting. The patient had been presenting to the hospital with similar recurrent symptoms since eight months of age.

**Figure 1:**
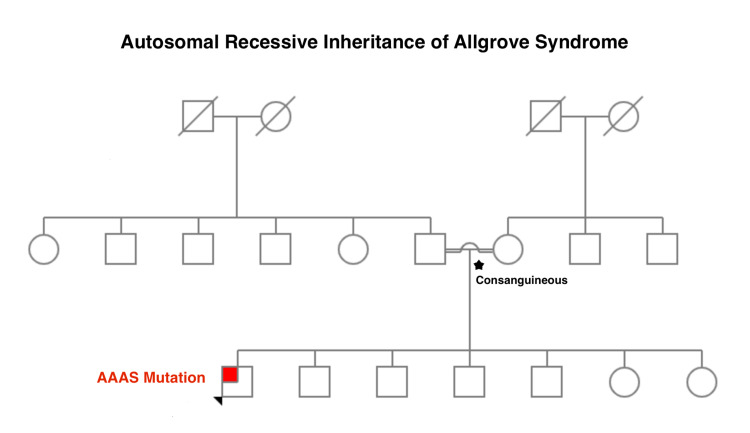
Pedigree of AAAS gene mutation. The arrow indicates our patient while the red mark indicates the AAAS gene mutation. The star denotes the consanguineous relationship of the patient's parents.

Furthermore, our patient has a current weight of 25.7 kg (< 3rd percentile) and height of 161 cm (5th percentile), with a BMI of 9.91 kg/m2. Our patient was also found to have a low blood sugar of 68 mg/dL manifested clinically as lethargy and easy fatiguability. Current medications included daily prednisolone 5 mg. Family history is notable for consanguinity and is insignificant despite having six other siblings as shown in the pedigree in Figure 1.

On examination, the patient appeared cachectic and had an unsteady gait. Further examination revealed mild conjunctivitis, hyperpigmentation of the gingiva and axilla. External genitalia examination revealed Prader stage 4 for pubic hair and Prader stage 3 for axillary hair upon inspection. Our patient had bilateral rigid cavovarus feet, which were more prominent on the right. Coleman block testing was negative. Vitals were notable for a blood pressure of 94/62 mmHg.

Intravenous fluids were initiated and an initial CXR and ECG were unremarkable. Early morning ACTH and cortisol levels were taken after five days of stopping prednisolone. Results revealed a cortisol level of 213 nmol/L (<270 nmol/L is considered low) and an ACTH level of 2.38 pmol/L (normal levels: 2.2 and 13.3 pmol/L). Manometry confirmed Achalasia type II. Laboratory results demonstrated in Table 1 shows high phosphate levels but low vitamin D levels.

**Table 1:** Laboratory parameters. WBC: white blood cells; CO2: carbon dioxide; INR: international normalized ratio.

Laboratory parameter	Value	Normal range
Hemoglobin	10.9 g/dL	13.5-17.5 g/dL
Hematocrit	35%	41%-53%
WBC	4.04 K/uL	4.5-11 K/uL
Creatinine	41 μmol/L	53-106 μmol/L
CO2	19 mEq/L	23-29 mEq/L
INR	1.24	0.8-1.1

Management plan included an elective admission for peroral endoscopic myotomy (POEM) with 50 mg of IV hydrocortisone 30-60 minutes prior to the procedure. Oral prednisolone was discontinued, and enoxaparin was stopped 12 hours prior to the procedure. The patient successfully underwent POEM with no complications. Postprocedural barium swallow was performed and ruled out any perforation or leakage. Postprocedural labs were insignificant. The patient was started on postprocedural cefazoline, prophylactic proton pump inhibitors, analgesia and antiemetics as needed as well as cholecalciferol 50,000 International Units (IU) once weekly for six weeks, then monthly. Moreover, the patient was scheduled for regular follow-up with Endocrinology and Nutrition. A gastrointestinal clinic visit was scheduled after two to four weeks for an upper gastrointestinal endoscopy as well as a repeat barium swallow.

## Discussion

AS or triple A syndrome is an autosomal recessive disorder with mutation of the AAAS gene. AAAS is located on chromosome 12q13, which is responsible for ALADIN protein [7]. ALADIN is involved in regulating multiple intracellular pathways such as signal transduction, nucleocytoplasmic transport, and transcription of RNA [8,9]. AS has an estimated prevalence of 1 in 1 million. However, it is suggested that the prevalence of this syndrome is inaccurate due to misdiagnosis and limited literature [8,9]. AS does not seem to be directly correlated with specific age, ethnicity, or sex and has varying degrees of severity on presentation [9]. AS classically presents with the triad of adrenal insufficiency, alacrimia, and achalasia in 70% of patients; however, patients may have a different collection of presenting symptoms [8]. Symptoms and signs of AS include alacrimia, ACTH resistant adrenal insufficiency, achalasia, dysphagia, autonomic disturbances, weight loss, hyperpigmentation, extrapyramidal dysfunction symptoms, recurrent infections, recurrent hypoglycemic episodes, neurologic deficits, mental retardation, hypotension, and orthostatic hypotension [10,11]. Thus, diagnosis of AS can be challenging due to the numerous presentations. Primarily, the diagnosis of AS is largely based on the detection of AAAS gene mutation in affected individuals. However, some patients with a deletion defect of the AAAS gene, whether partial or complete may present with a mutation non-detection [12].

In Saudi Arabia, consanguinity is common, as a result, there is a higher risk of multiple inherited diseases and adrenal gland disorders. In a review article done by Al-Jurrayan et al. [13] on primary adrenal insufficiency (PAI) etiologies in 125 patients, AS only compromised 1.6% of the etiologies of PAI. Thus, AS is rarely detected and can be confused with other PAI etiologies. In our case, our patient underwent multiple hospital visits without a definitive diagnosis. This led to his poor health status upon presentation. Early detection of AS is a crucial aspect of the disease management, and early interventions might minimize the life-threatening complications of adrenal insufficiency [13,14]. 

Treatments and procedures of AS are comparatively similar among the literature. The treatment is mainly conservative and revolves around symptomatic management. Artificial tears eyedrops are sufficient to treat symptoms of alacrimia and might play a role in preventing ophthalmic complications [9]. While for achalasia treatment modalities differ with the varying degrees of dysphagia and esophageal patency. Mild to moderate achalasia might be responsive to pneumatic endoscopic esophageal dilation and pharmacotherapy such as nifedipine, while severe achalasia might require esophageal myotomy procedures such as Heller’s myotomy [14]. Lastly, adrenal insufficiency is variable between patients and can present as partial adrenal insufficiency (such as our case) or as severe as an adrenal crisis. Thus, treatments for adrenal insufficiency are highly dependent on the adrenal gland function. Treatment for severe adrenal insufficiency is usually oral glucocorticoids such as prednisolone or hydrocortisone [10]. In our case, however, the patient had partial adrenal insufficiency with low but relatively normal cortisol and the discontinuation of prednisolone was chosen as the best route of treatment. 

## Conclusions

AS is a rare inherited disorder that presents classically as a triad of alacrimia, achalasia and adrenal insufficiency. However, this triad is not seen in all patients, and can pose a challenging diagnosis when absent. Genetic analysis and detection of AAAS gene mutation is the cornerstone of diagnosis. Delayed detection may result in multiple hospital admissions and life-threatening complications. More data and research are needed to aid in expanding the knowledge about AS in the literature.
